# Granulocytic Myeloid-Derived Suppressor Cell Exosomal Prostaglandin E2 Ameliorates Collagen-Induced Arthritis by Enhancing IL-10^+^ B Cells

**DOI:** 10.3389/fimmu.2020.588500

**Published:** 2020-11-30

**Authors:** Xinyu Wu, Dongwei Zhu, Jie Tian, Xinyi Tang, Hongye Guo, Jie Ma, Huaxi Xu, Shengjun Wang

**Affiliations:** ^1^ Department of Laboratory Medicine, The Affiliated People’s Hospital, Jiangsu University, Zhenjiang, China; ^2^ Department of Immunology, Jiangsu Key Laboratory of Laboratory Medicine, School of Medicine, Jiangsu University, Zhenjiang, China

**Keywords:** granulocytic myeloid-derived suppressor cells, exosomes, prostaglandin E2, collagen-induced arthritis, IL-10^+^ Breg cells

## Abstract

The results of recent studies have shown that granulocytic-myeloid derived suppressor cells (G-MDSCs) can secrete exosomes that transport various biologically active molecules with regulatory effects on immune cells. However, their roles in autoimmune diseases such as rheumatoid arthritis remain to be further elucidated. In the present study, we investigated the influence of exosomes from G-MDSCs on the humoral immune response in murine collagen-induced arthritis (CIA). G-MDSCs exosomes-treated mice showed lower arthritis index values and decreased inflammatory cell infiltration. Treatment with G-MDSCs exosomes promoted splenic B cells to secrete IL-10 both *in vivo* and *in vitro*. In addition, a decrease in the proportion of plasma cells and follicular helper T cells was observed in drainage lymph nodes from G-MDSCs exosomes-treated mice. Moreover, lower serum levels of IgG were detected in G-MDSCs exosomes-treated mice, indicating an alteration of the humoral environment. Mechanistic studies showed that exosomal prostaglandin E2 (PGE2) produced by G-MDSCs upregulated the phosphorylation levels of GSK-3β and CREB, which play a key role in the production of IL-10^+^ B cells. Taken together, our findings demonstrated that G-MDSC exosomal PGE2 attenuates CIA in mice by promoting the generation of IL-10^+^ Breg cells.

## Introduction

Rheumatoid arthritis (RA) is a systemic autoimmune disease characterized by chronic inflammation in the synovium. Collagen-induced arthritis (CIA) is a well-established experimental model of human RA in mice, which exhibit severe swelling of the paws, synovial hyperplasia and joint ankylosis after type II collagen immunization (1–3). These phenomena are the result of the infiltration of lymphocytes into the synovium and the production of collagen-specific IgG autoantibodies by B cells (4). Recently, a new subset of B cells termed regulatory B cells (Breg cells) were identified that can secrete interleukin-10 (IL-10) to inhibit the production of proinflammatory cytokines and restrain excessive immune responses (5, 6). The adoptive transfer of Breg cells has been reported to suppress the development of arthritis (7). Additionally, B cell-derived IL-10 is necessary for protecting NOD mice from Type 1 diabetes (T1D), as the infusion of NOD-IL-10^−/−^ B cells has no effect on disease improvement (8). Therefore, the use of Breg cells may be an effective means of treating RA in the future.

Myeloid derived suppressor cells (MDSCs) are heterogeneous immature myeloid cells that possess a strong ability to suppress immune responses (9, 10). According to their morphological characteristics and the expression of Gr-1, murine MDSCs are divided into two major populations: granulocytic MDSCs (G-MDSCs) and monocytic MDSCs (M-MDSCs) (11, 12). Both cell types can expand and accumulate under pathological conditions, including tumor formation, inflammation and pathogen infection (13, 14). In addition these cells can express immunosuppressive factors such as arginase-1 (Arg-1), inducible nitric oxide synthase (iNOS), transforming growth factor-β (TGF-β) and cyclooxygenase-2 (COX-2) (15). COX2 is an inducible enzyme that can be activated in variety of cells under specific conditions (16). As COX2 can convert arachidonic acid (AA) into prostaglandin E2 (PGE2), the level of PGE2 is typically used as an indicator of COX2 activity (17). PGE2 is a proinflammatory mediator produced by cancer and myeloid cells that acts on G-protein-coupled receptors (18, 19). MDSCs have been reported to express high levels of COX2 and are a major source of PGE2. This positive feedback loop between PGE2 and COX2 plays important role in the function and stability of MDSCs (20, 21). Studies have shown that PGE2 can regulate Th2-mediated cytokine spectrum, especially with respect to promoting IL-10 production (22, 23). PGE2 can also exert anti-inflammatory activities on macrophages and dendritic cells (24, 25). These effects are highly correlated with the inhibition of glycogen synthesis kinase 3 (GSK3) induced by PGE2. GSK3 is a ubiquitous serine/threonine kinase that has been shown to be a convergence point for many signaling pathways, with GSK3 being able to phosphorylate over 50 substrates to regulate cellular function (26). Furthermore, the effective use of GSK3 inhibitors to treat CIA has been reported due to their ability to alleviate joint swelling and eliminate histologically graded damage (27).

Exosomes are nanosized vesicles produced by multivesicular bodies (MVBs) that harbor multiple membrane proteins, such as CD9, CD81, and CD63 (28). Small molecules, such as water-soluble proteins, nucleic acids, and lipids can be transported from donor to recipient cells through exosomes, allowing for the exchange of substances and information between donor and recipient cells (29). Exosomes exhibit better stability than the parent cells in the treatment of diseases. Furthermore, studies have shown that the exosomes of immature DCs treated with immunoregulatory cytokines can inhibit inflammation and alleviate the course of CIA in the rat footpad model of delayed hypersensitivity (30). Therefore, exosomes may be used in potential therapies for arthritis and other autoimmune diseases in the future (31).

In the present study, we showed that G-MDSCs exosomes (G-exo) could attenuate the disease process of murine CIA. Furthermore, G-exo could promote IL-10^+^ Breg cell generation *in vivo* and *in vitro*, which was primarily associated with exosomal PGE2. Taken together, the results of the present study demonstrated that G-MDSC exosomes are a potentially novel mediator for the treatment of CIA mice.

## Materials and Methods

### Mice

DBA1/J mice (8–10 weeks old, male) were purchased from the Shanghai Laboratory Animal Center (Shanghai, China), and C57BL/6 mice (6–8 weeks, male) were purchased from the Jiangsu University Animal Center (Zhengjiang, China). All animal experiments performed in the present study were approved by the Jiangsu University Animal Ethics and Experimentation Committee.

### Induction and Assessment of Arthritis

Briefly, the DBA1/J mice were immunized by injecting 100 μg of emulsions that were acquired by bovine type II collagen (CII; Chondrex, WA, USA) emulsified with an equal volume of complete Freund’s adjuvant (CFA, Sigma-Aldrich, St. Louis, MO, USA). On day 21, the mice received a secondary immunization with a booster emulsion prepared with CII and incomplete Freund’s adjuvant near the primary injection site. To determine the effects of the G-exo treatment, mice received 100 μg of G-exo on days 18 and 24 after the first immunization. From day 21 on, mice were scored for signs of arthritis every 3 days. Each paw was evaluated and scored individually using a 0 to 4 scoring system as previously described (27).

### Histopathologic Examination

Mice were sacrificed on day 42, and murine joint tissue specimens were obtained and fixed in 10% phosphate-buffered formalin for 3 days. Tissue sections (4-μm-thick) were stained with H&E to examine morphological features and perform histologic arthritis scoring.

### Isolation of G-MDSCs

Tumor –bearing mice were established with the Lewis lung adenocarcinoma cell line. G-MDSCs were harvested from mouse spleens using G-MDSC isolation kits (Miltenyi Biotec, Cologne, DE) according to the manufacturer’s protocol. The purity was evaluated by measuring the expression of Ly-6G and CD11b *via* flow cytometry (FCM).

### Extraction and Identification of Exosomes

G-MDSCs were first cultured in plates (in R1640 medium supplemented with 10% fetal bovine serum ultracentrifuged at 100,000*g* for 16 h at 4°C) at 37°C under an atmosphere with 5% CO_2_. After 16 h, the culture supernatant was obtained. Subsequently, the cultures were centrifuged to remove debris as follows: 500 × g for 10 min at 4°C, 1,000*g* for 10 min and 10,000*g* for 30 min. Then, the supernatants were filtered through 0.22-μm pore filters (Millipore, Billerica, MA, USA). Exosomes were precipitated using an exosome extraction kit (System Biosciences, Palo Alto, CA, USA), dissolved in PBS and then stored at −80°C. During the purification of celecoxib-treated G-exo, the G-MDSCs used to extract exosomes were cultured in the presence of graded doses of celecoxib (Pfizer Inc., La Jolla, CA, USA). Subsequently, the purified G-exo were fixed and examined by transmission microscopy (Tecnai-12; Philips, Amsterdam, Netherlands) as previously described. The protein concentrations were determined by using a Micro BCA protein assay kit (Beijing ComWin Biotech, Beijing, China). In this study, we also prepared neutrophil-derived exo (Neu exo), which served as control for G-MDSC exo. The concentration and size distribution of the G-exo were measured *via* nanoparticle tracking analysis (NTA). The expression of the exosomal marker CD63, CD9, and the negative marker calnexin was measured by Western blot analysis.

### Isolation and In Vitro Culturing of B Cells

For *in vitro* experiments, CD19^+^ B cells were isolated from spleens using mouse CD19 microbeads (Miltenyi Biotec). Then, B cells were cultured in medium supplemented with 10 µg/ml LPS (Sigma-Aldrich) alone or together with G-exo.

### Exosomes Labeling and Uptake

Exosomes were labeled with PKH67 (Sigma-Aldrich) according to manufacturer’s instructions. Isolated B cells were first incubated with labeled exosomes for 6 h before being washed with exosome-depleted FBS and then pelleted by centrifugation for 5 min in 400 × g. The pelleted cells were then resuspended, after which red fluorescent dye PE (Bio-legend) was added to the cell suspension for 4 min. Subsequently, the reaction was stopped by the addition of an equivalent volume of exosome-depleted FBS, after which the cells were washed with PBS, fixed with 4% paraformaldehyde for 15 min, and then mounted with Hoechst nuclear stain.

### Flow Cytometry Analysis

Single cell suspensions were immunostained with various combinations of fluorescent dye-conjugated antibodies against the following proteins: CD19, CD4, CXCR5, PD-1, CD138, and B220 (eBioscience, San Diego, CA, USA). For intracellular cytokine staining, single cell suspensions were simulated with 50 ng/ml of phorbol myristate acetate (Sigma-Aldrich), 1 µg/ml of ionomycin (Enzo, Farmingdale, NY, USA) and 2 µg/ml of monensin (Enzo) for 5 h. Then, the anti-IL-10 mAb (eBioscience) used to for intracellular staining of IL-10 according to the manufacturer’s instructions, and the stained cells were analyzed by flow cytometry using a FACS Calibur instrument (BD Biosciences, San Jose, CA, USA).

### Quantitative Reverse Transcription PCR (RT-qPCR)

To assess gene expression in B cells, we first extracted total RNA from samples using TRIzol reagent (Invitrogen, Carlsbad, CA, USA). Then, cDNA was synthesized with a ReverTraAce qPCR RT kit (Toyobo, Osaka, Japan). The expression of IL-10 was assessed using the cDNA samples for RT-qPCR analysis based on SYBR green detection method (Bio-Rad, Hercules, USA).The sequences of the primers used are as follows: IL-10, 5-GGTTGCCAAGCCTTATCGGA-3 (forward), 5-ACCTGCTCCACTGCCTTGCT-3 (reverse).

### ELISA

The levels of total IgG in murine serum samples and IL-10 in culture supernatant were measured using ELISA Ready SET-Go kits (eBioscience) following the manufacturer’s protocol. The concentration of prostaglandin E2 (PGE2) was assessed using a Prostaglandin E2 ELISA Kit-Monoclonal (Cayman Chemical, Ann Arbor, MI) following the manufacturer’s instructions. This assay is based on the competition between PGE2-acetylcholinesterase conjugate for a limited amount of PGE2 monoclonal antibody. PGE2 was quantified using the equation obtained from the standard curve plot. Chick collagen type 2 was coated on the plate overnight at 4°C. The plates were washed with 0.05% Tween-20 in PBS and then blocked with 5% BSA for 1 h. Then the plates were washed for three times and added into diluted serum. The plates were incubated at 37°C for 1 h. Subquently, the plates were washed and added with HRP-labeled lgG antibodies. Finally, OPD peroxidase substrate was added into plates. The absorbance was measured at 450 nm.

### Western Blot Analysis

Proteins extracted from cells were prepared as described previously. Then, the lysates were separated by 12% SDS-PAGE and transferred onto immobilon polyvinylidene difluoride (PVDF) membranes (Bio-Rad), which were then blocked with 5% BSA in Tris-buffered saline with Tween 20. The membranes were then incubated with specific rabbit antibodies against phosphorylated (p)-GSK-3β (Santa Cruz Biotechnology, Texas, USA*)*, total (t)- GSK-3β (Wanleibio, Shenyang, China), phosphorylated (p)-cAMP response element binding protein (CREB) (Wanleibio, Shenyang, China) and total (t)-CREB (Wanleibio, Shenyang, China) followed by an incubation with the secondary HRP-conjugated goat anti-rabbit IgG (CST, Danvers, MA, USA) according to manufacturer’s protocol. Finally, chemiluminescent detection reagent (Champion Chemical, CA, USA) was used to visualize the bands on the PVDF membranes.

### Statistical Analysis

All data were analyzed with a two-tailed Student’s t-test or one-way ANOVA using SPSS 16.0. When the p value was less than 0.05, differences were considered statistically significant.

## Results

### Extraction and Identification of G-MDSCs Exosomes

**Figure 1:**
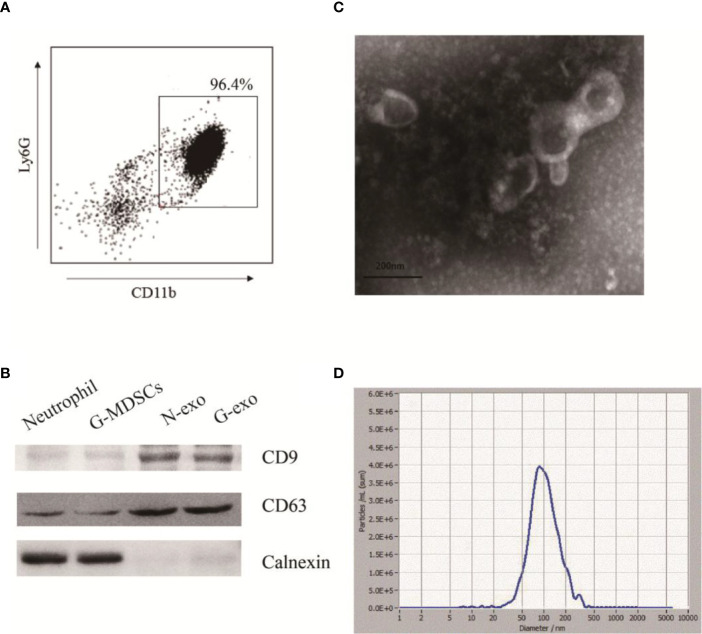
Extraction and identification of G-exo. **(A)** G-MDSCs were sorted from mouse spleens using immunomagnetic beads. The expression of Ly-6G and CD11b was analyzed by flow cytometry. **(B)** The expression of CD63, CD9 and calnexin was assessed by Western blot analysis. **(C)** Representative transmission electron micrograph of G-exo (scale bar = 200 nm). **(D)** Particle size distribution of G-exo analyzed by nanoparticle tracking analysis (NTA). The presented data are from one of three independent experiments.

G-MDSCs were isolated from the spleens and analyzed by flow cytometry. The results showed that the G-MDSC purity was greater than 95%, which met the needs for follow-up experiments (**Figure 1A**). Subsequently, G-exo were extracted by differential centrifugation followed by the use of an exosome extraction kit according to the manufacturer’s instructions. To identify the specific proteins expressed on exosomes, the extracted G-exo were evaluated by Western blot analysis. The results showed that CD63 was expressed in G-exo, whereas calnexin was not detected (**Figure 1B**). Further characterization by transmission electron microscopy and nanoparticle tracking analysis (NTA) indicated that the exosomes were saucer-like bilayer membrane vesicles with a particle size distribution peak of 99.6 nm (**Figures 1C, D**).

### G-MDSCs Exosomes Attenuate the Development of CIA in Mice

**Figure 2:**
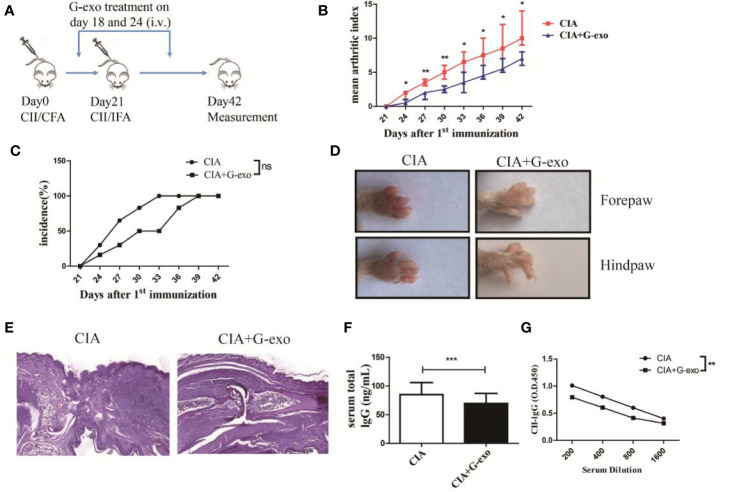
G-exo attenuate collagen-induced arthritis in mice. **(A)** DBA1/J mice were immunized with CII/CFA on day 0 and boosted with CII/IFA on day 21. The treatment groups were intravenously injected with 100 μg of G-exo (CIA+G-exo) or phosphate buffered saline (control CIA) on days of 18 and 24. **(B)** The mean arthritis index was assessed once every three days after day 21 for the G-exo-treated or control mice according to the criteria for evaluation. **(C)** Incidence was assessed once every three days after day 21 for the G-exo-treated or control mice**. (D)** Images of hind paws and forepaws of the G-exo-treated or control mice. **(E)** Hind paws obtained from G-exo-treated or control mice were analyzed by H&E histologic examination. **(F)** Serum levels of total IgG were measured by ELISA. **(G)** Serum anti-CII antibody levels were measured by ELISA. Bar graphs show the means ± SD, *P < 0.05; **P < 0.01; ***P < 0.001; ns indicates no significance, (n = 6).

To test whether G-exo can attenuate murine CIA, DBA1/J mice were administered an i.p. injection of G-exo (100 μg/mouse/injection) on days 18 and 24 after the first immunization. We observed that G-exo-treated mice were less susceptible to CIA than mice in the control group. First, the arthritis onset and disease progression were analyzed. As shown in **Figure 2B**, the mean arthritis index of G-exo-treated mice was lower than that observed in control mice. Although there was no significantly difference, CIA group mice had been completely suffered at 33 days, while mice treated with G-exo did not become fully ill until 39 days (**Figure 2C**). Second, the swelling caused by collagen stimulation in paws was strikingly reduced by G-exo treatment, while this change was not observed in the control group mice (**Figure 2D**). Third, histologic hind paw examinations revealed that substantial inflammatory cells infiltrated the articular cavity of control mice, which was accompanied by synovial cell proliferation and injury of the articular cartilage. In contrast, G-exo-treated arthritic mice showed significant improvement in the joint injury (**Figure 2E**). Furthermore, markedly lower levels of total IgG and anti CII antibodies were observed in the serum samples of G-exo-treated mice compared to that observed in the control mice (**Figures 2F, G**). Taken together, these results suggested that G-exo can attenuate collagen-induced arthritis, indicating their potential therapeutic effect.

### G-MDSCs Exosomes Increase the Proportion of IL-10^+^ B Cells but Decrease That of Plasma Cells and Follicular Helper T Cells in CIA

**Figure 3:**
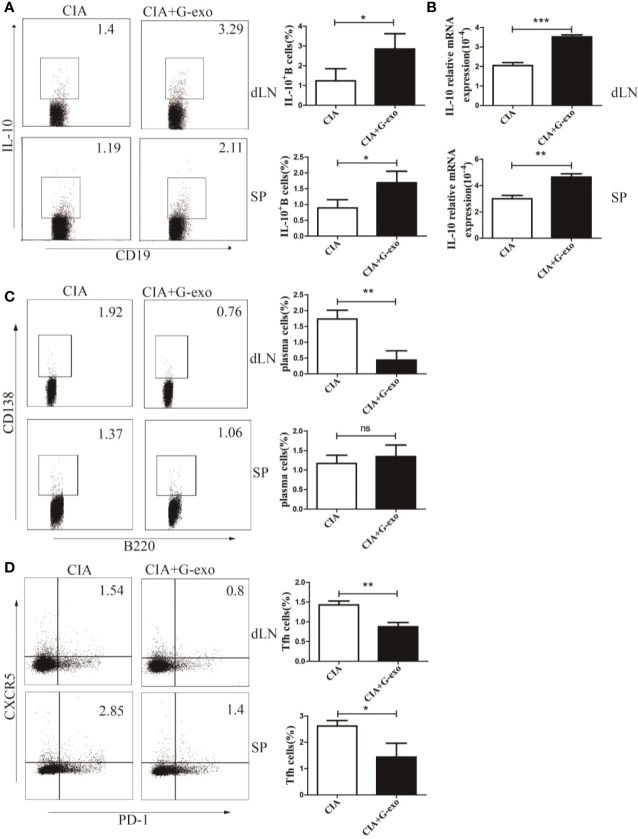
G-exo increase IL-10^+^ B cells but decreases the proportion of plasma cells and follicular helper T cells in CIA. **(A)** Proportions of interleukin-10 (IL-10)–producing B cells in drainage lymph nodes (dLNs) and spleens from CIA mice treated with G-exo (CIA+G-exo) or control mice (CIA) were analyzed by FCM. **(B)** The level of IL-10 mRNA in dLNs (up) and spleens (down) was determined by RT-qPCR. **(C)** The proportion of B220^-^CD138^+^ plasma cells in dLNs and spleens was analyzed by FCM. **(D)** The proportion of CD4^+^CXCR5^+^PD-1^+^ follicular helper T cells in dLNs and spleens was analyzed by FCM. Bar graphs show the means ± SD. *P < 0.05; **P < 0.01; ***P < 0.001; ns indicates no significance.

Considering the effect of G-exo on CIA mice described above, we next assessed whether G-exo treatment may have regulate the humoral immune response in arthritic mice. As shown in **Figure 3A**, the proportions of IL-10^+^ B cells in the spleens and drainage lymph nodes (dLNs) of arthritic mice treated with G-exo were increased compared with that observed in control mice. The level of mRNA IL-10 was also upregulated in the spleens and dLNs in G-exo-treated CIA mice (**Figure 3B**). In addition, further results revealed that mice treated with G-exo had a decreased proportion of B220^-^CD138^+^ plasma cells in the dLNs, while no significant difference was observed in the spleens (**Figure 3C**). The proportion of CD4^+^CXCR5^+^PD-1^+^ follicular helper T (Tfh) cells was decreased in the spleens and dLNs of arthritic mice treated with G-exo (**Figure 3D**). Thus, our data indicated that G-exo promoted IL-10^+^ B cells generation but inhibited Tfh and plasma cells, which may contribute to the remission of arthritis.

### G-MDSCs Exosomes Promote the Generation of IL-10–Producing B Cells In Vitro

**Figure 4:**
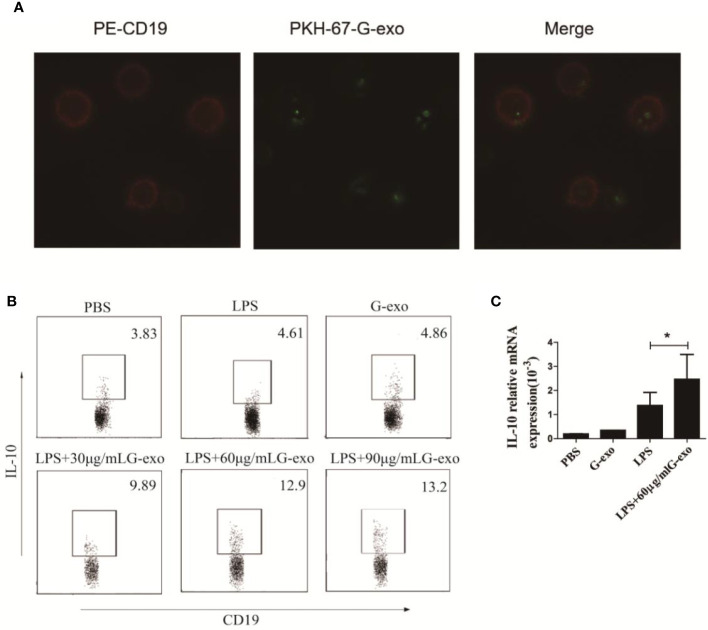
G-exo promote the generation of IL-10^+^ B cells *in vitro*. **(A)** Representative confocal microscopy images of B cells treated with PKH-67 (green)-tagged G-exo for 6 h. Cells were stained with the PE-anti CD19 mAb for the membrane. **(B)** Splenic B cells were cultured with LPS (10 μg/ml), G-exo (30 μg/ml), or with a combination of LPS and different concentrations of G-exo for 48 h. IL-10–producing B cells were analyzed by FCM. **(C)** Levels of IL-10 mRNA were determined by RT-qPCR. Bar graphs show the means ± SD. *P < 0.05. The presented data are from one of three independent experiments.

Based on the regulatory effect of G-exo on arthritic mice, we examined the role of G-exo in B cells *in vitro*. Splenic B cells were first cocultured with PKH-67–tagged G-exo for 6 h. Subsequently, the membranes of cultured B cells were stained with PE dye. As shown in **Figure 4A**, the images revealed that G-exo could be taken up by B cells, as determined by confocal microscopy analysis. Next, B cells were cultured with LPS (10 μg/ml), G-exo (30 μg/ml), or with a combination of LPS and different concentrations of G-exo for 48 h. IL-10–producing B cells were analyzed by FCM, and the combination (32) of G-exo and LPS was observed to increase the proportion of IL-10–producing B cells *in vitro* (**Figure 4B**). These results further confirmed that the level of IL-10 mRNA expression was increased by the combination treatment of G-exo and LPS (**Figure 4C**). These findings confirmed that G-exo promoted the production of IL-10–producing B cells *in vitro*.

### Exosomal Prostaglandin E2 Produced by G-MDSCs Is Key for the Production of IL-10–Producing B Cells

**Figure 5:**
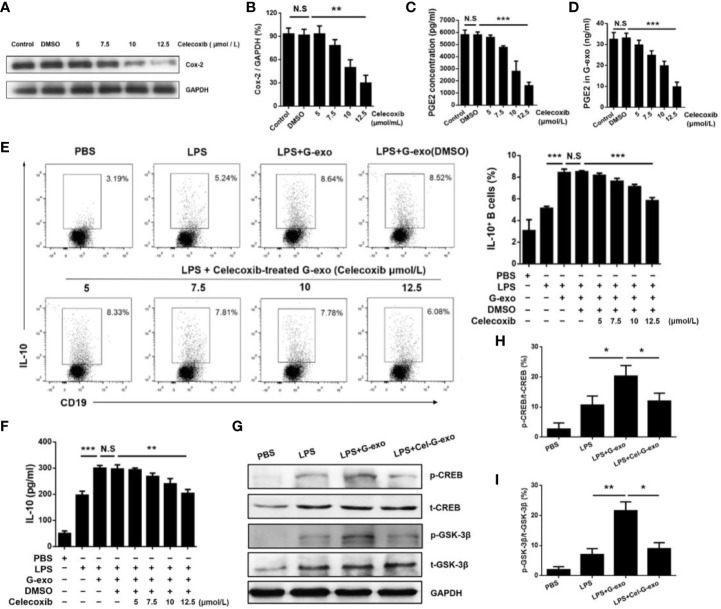
G-MDSC exosomal PGE2 promotes IL-10^+^ B cells**(A)** COX-2 expression in G-MDSCs was detected after treatment with the COX-2 inhibitor celecoxib. **(B)** The ratio of COX2 and GAPDH in each group was statistically analyzed. **(C)** The level of PGE2 in the G-MDSC culture supernatant treated with celecoxib was detected by ELISA. **(D)** The level of PGE2 in the G-exo treated with celecoxib was detected by ELISA. **(E)** IL-10–producing B cells after treatment with celecoxib-treated G-exo in the present of LPS were analyzed by flow cytometry. **(F)** The level of IL-10 in culture supernatant after treatment with celecoxib-treated G-exo was detected by ELISA. **(G)** The p-GSK-3β, T-GSK-3β, p-CREB, and T-CREB were detected by Western blot analysis. **(H)** The ratio of p-CREB and T-CREB in each group was statistically analyzed. **(I)** The ratio of p-GSK-3β and T-GSK-3β in each group was statistically analyzed. Bar graphs show the means ± SD. *P < 0.05; **P < 0.01; ***P < 0.001; ns: indicates no significance.

G-MDSCs were cultured in medium alone or with graded doses of celecoxib, a COX-2 inhibitor, for 16 h. First, the celecoxib was shown to have no effect on the number and viability of G-MDSCs (**Supplemental Figure 1**). The results showed that celecoxib could effectively reduce the expression of COX-2 in a dose-dependent manner (**Figures 5A, B**). As COX-2 is a biosynthetic enzyme involved in the synthesis of PGE2, the levels of PGE2 in the MDSC supernatant and exosomes in the celecoxib treatment group were significantly lower than that observed in control or dimethyl sulfoxide (DMSO) groups, indicating that celecoxib reduced the level of PGE2 in G-MDSC exosomes (**Figures 5C, D**). As shown in **Figure 5E**, G-exo treated with celecoxib could no longer promote IL-10 production by B cells in the presence of LPS. This result was further confirmed by the levels of IL-10 being downregulated in G-exo treated with celecoxib group (**Figure 5F**).

**Figure 6:**
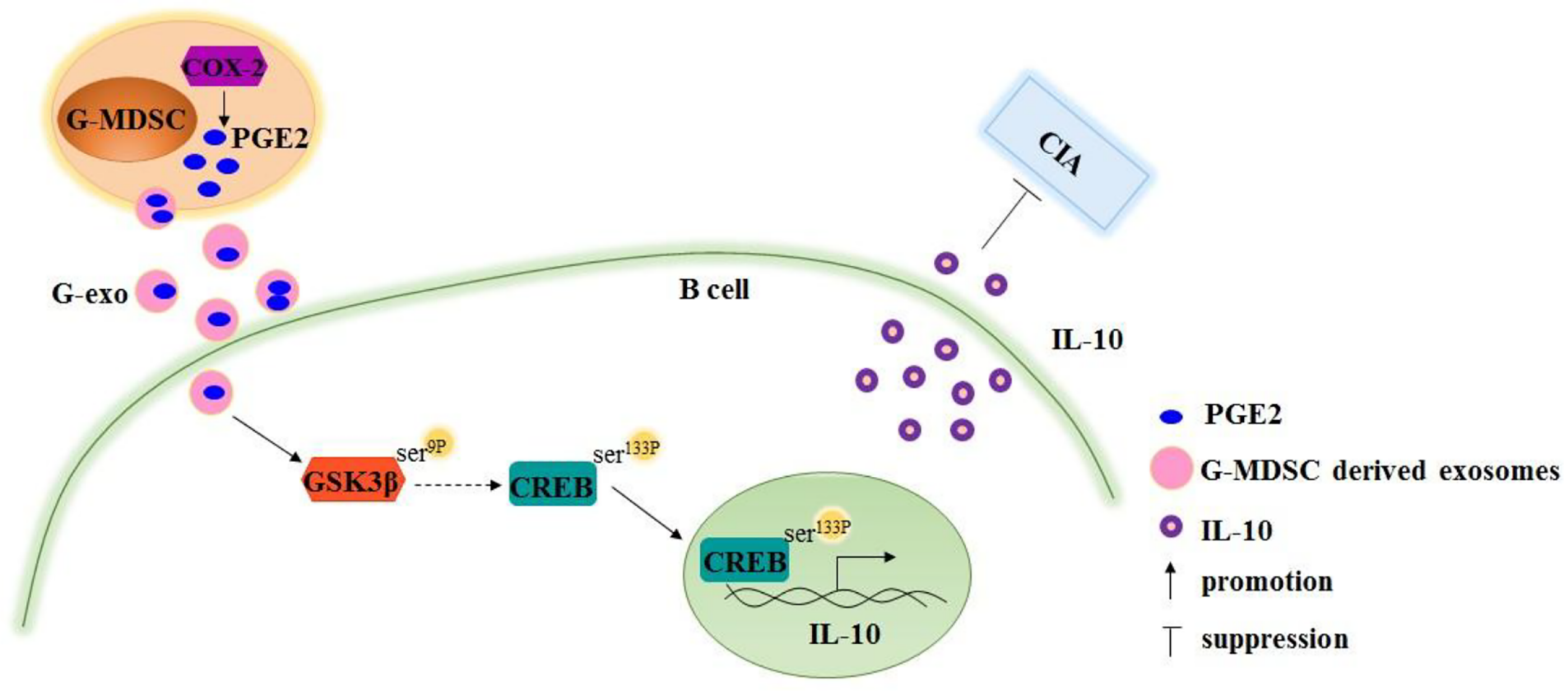
Schematic image demonstrating that Granulocytic myeloid-derived suppressor cell exosomal prostaglandin E2 ameliorates collagen-induced arthritis by inducing IL-10^+^ B cells production *via* affecting GSK-3β and CREB phosphorylation.

The GSK3 signaling pathway has been reported to regulate IL-10 expression, and PGE2 treatment leads to GSK-3β phosphorylation (28, 29). Therefore, we tested the activation of GSK-3β signaling molecules in B cells treated with G-exo or G-exo treated with celecoxib. We observed that G-exo upregulated GSK-3β phosphorylation at Ser9 and CREB at Ser133 in B cells, while GSK-3β and CREB phosphorylation was not significantly altered in G-exo treated with celecoxib (**Figures 5G–I**). These findings suggest that G-MDSC exosomal PGE2 may promote IL-10 production by B cells, which may involve the GSK-3β signal pathway (**Figure 6**).

## Discussion

In recent years, most studies established the prominent role of MDSCs in the regulation of immune responses during autoimmune pathology. Park et al. showed that adoptive transfer of MDSCs could decrease the number of Th1 and Th17 cells, both of which were widely considered as the important mediators in the pathogenesis of RA. However, they also found that transfer with MDSCs increased the number of Treg cells in spleen of CIA mice (32). Considering the types of MDSCs, sources of MDSCs and cell dose of MDSCs used in adoptive transfer, the exactly regulatory effect of MDSCs on various effector CD4^+^ T cells populations in the CIA pathogenesis remains to be further researched. In addition, the effect of MDSCs on B cells is unclear. Exosomes are a type of membrane vesicle that can be transported to target cells, affecting the behavior and activity of the recipient cells. Zoller et al. have reported the immunomodulatory effect of MDSCs derived exosomes in mouse model of autoimmune alopecia areata (33). Wang et al. also showed that G-exo could be used as a therapeutic approach because these exosomes could reduce DSS-induced-colitis (34). Our previous studies have already shown that the therapeutic effect of G-exo on CIA mice was by inhibiting the differentiation of Th1 and Th17 cells (35). Therefore, we further explored the role of G-exo on B cells in CIA mice. In the current study, the administration of G-exo indeed improved joint injury and slowed the disease process in CIA mice, and we noticed the decreased level of antibody in G-exo treated CIA mice. However, Crook et al. reported that PMN-MDSCs had no effect on B cell response but MO-MDSCs inhibited B cell proliferation and antibody production *via* iNOS and PGE2 in a contact-dependent manner (36). On the one hand, the reason for this discrepancy is due to the different sources of MDSCs, because G-MDSCs used in this experiment were isolated from tumor-bearing mice while MDSCs used in Crooks’ experiment were isolated from CIA mice. On the other hand, it is attributed to different ways of action. In addition, we also discovered that G-exo could influence the frequency of plasma cells and Tfh cells. For the first time, we demonstrated that G-exo could reduce the proportion of Tfh cells in spleen and draining lymph nodes. Majority studies focused on the changes of Tfh cells in spleen of mice. However, MDSCs derived exosomes were more enriched in draining lymph nodes cells and were retained in skin-infiltrating leukocytes of AA mice for up to 48 h after tail vein injection (33). In view of the characteristic of MDSCs derived exosomes, it was essential to detect the effect of exosomes on lymph node cells in some autoimmune diseases. Over the past decade, B10 cells have been described as a functional subtype of immunosuppressive Bregs in the B cell population, characterized by the production of IL-10 in humans or mice. Park et al. reported MDSCs induced the expansion of regulatory B cells and ameliorated systemic lupus erythematosus severity in mice (37). Similarly, we also found G-exo could not only upregulate the proportion of IL-10–producing B cells in CIA mice but also induce IL-10^+^ B cells *in vitro*. As previous studies reported, adoptive transfer of regulatory B cells into CIA models could ameliorate disease severity. Thus, our experiments provided a potential pathway of action for G-exo in the therapeutic treatment of CIA. However, the spleen was the predominant reservoir of B10 cells in mice. B10 cells and B10 precursor cells were rare within the lymph nodes and peripheral blood (38). Another guess had been put forward that immature B cells firstly differentiated into transitional B cells through Toll-like receptor, cytokines stimulation and CD40 activations. Then, the transitional B cells differentiated into Breg cells (39). This suggests that Breg cell development is no longer limited to the spleen. Hence, the origin of B10 cells needs to be further researched.

Many previous studies have shown the biological effects of PGE2 and other cAMP-increasing substances on the cytokine profiles of macrophages and dendritic cells, which resulted in the suppression of inflammatory cytokines and enhanced levels of the anti-inflammatory cytokine IL-10 (40, 41). COX-2 is a key enzyme of PGE2 synthesis, and celecoxib is a selective COX-2 inhibitor. Therefore, celecoxib was chosen to inhibit the expression of PGE2 in G-MDSCs. We observed that G-exo contained a high concentration of PGE2, and treatment with celecoxib downregulated the level of PGE2 in G-exo. Interestingly, when PGE2 in G-exo was blocked by celecoxib, the ability of G-exo to induce IL-10^+^ Breg cells was inhibited, suggesting that PGE2 is key factor for IL-10^+^ Breg cell generation. Majority studies implied that the levels of PGE2 were higher in serum and synovial of arthritis patients and mice. However, Frolov et al. reported that PGE2 acted as important negative regulators in collagen antibody induced arthritis(CAIA) (42). Another study also showed that PGD2 prevent CIA from joint inflammation and destruction (43). Thus, our experiment aimed to imply that despite PGE2 were enriched in articular tissue or serum during the development of CIA, they could also play anti-inflammatory role in this process.

It has also been reported that PGE2 together with TLR agonists can promote the production of IL-10 in macrophages by activating the CREB-regulated transcriptional coactivator (44). Furthermore, in the PI3K/AKT pathway, AKT attenuates the activity of GSK3 by phosphorylation, which regulates the function of CREB (45). To elucidate a possible molecular mechanism by which G-MDSC exosomal PGE2 promotes Breg generation, we examined the levels of the signaling molecules GSK-3β and CREB. In the present study, we observed that G-exo enhanced the phosphorylation of GSK-3β at Ser9 and CREB at Ser133 in B cells, and the phosphorylation decreased when G-MDSC exosomal PEG2 was blocked by celecoxib. This finding suggests that the role of G-exo on Breg cells is mediated by the GSK-3β pathway. However, we did not observe the phenotype of IL-10–producing B cells in our studies, which are consistently characterized by the unique phenotype CD1d^hi^CD5^+^ (46). In this study, 100 μg G-exo was injected into mice *via* the tail vein, which was consistent with most studies. It is still to be further explored if the therapeutic effect will increase with the amount of G-exo. However, Smyth et al. found that Balb/C mice were injected intravenously with 400 μg 4T1 cell-derived exosomes, and the mice rapidly developed dyspnea (47). Thus, the application of exosomes in clinical treatment is still limited by the dose, mode and toxicity of exosomes (48). But as shown, we believe it could still better complement the therapeutic effect of G-exo on CIA. Moreover, previous studies have shown that exosomes could be taken up by B cells, while PGE2 played a role by recognizing EP receptors on target cells, raising the question of whether PGE2 regulates the expression of PI3K by direct or indirect means.

In summary, we identified G-MDSCs derived exosomes as a potential mediator in the treatment of CIA mice. G-MDSCs derived exosomes mediate high levels of PGE2, subsequently promoting the generation of Breg cells with immunosuppressive function. These data further improve our understanding of the application of G-MDSCs derived exosomes in autoimmune arthritis.

## Data Availability Statement

The original contributions presented in the study are included in the article/**Supplementary Material**. Further inquiries can be directed to the corresponding author.

## Ethics Statement

The animal study was reviewed and approved by Jiangsu University Animal Ethics and Experimentation Committee.

## Author Contributions

XW, DZ, and HG performed experiments. JT, XT, and JM carried out the data analysis. SW, JT, and HX designed the experiments. XW and SW wrote and edited the manuscript. All authors contributed to the article and approved the submitted version.

## Funding

This work was supported by the National Natural Foundation of China (Grant No. 31470881) and Jiangsu Province’s Key Medical Talents Program (Grant No. ZDRCB2016018).

## Conflict of Interest

The authors declare that the research was conducted in the absence of any commercial or financial relationships that could be construed as a potential conflict of interest.

## Abbreviations

G-MDSCs, granulocytic-myeloid derived suppressor cells; RA, rheumatoid arthritis; CIA, collagen-induced arthritis; CII, type II collagen; IL-10, interleukin-10; COX-2, cyclooxygenase-2; PGE2, prostaglandin E2; glycogen synthesis kinase-3β, GSK-3β; CREB, cAMP response element binding protein; Breg, regulatory B cells; Tfh, follicular helper T.
