# Supplementary material for: Granulocytic Myeloid-Derived Suppressor Cell Exosomal Prostaglandin E2 Ameliorates Collagen-Induced Arthritis by Enhancing IL-10+ B Cells
Source: Front Immunol. 2020 Nov 30;11:588500. doi: 10.3389/fimmu.2020.588500 (PMC7734343; doi:10.3389/fimmu.2020.588500)
Supplement: Supplementary file 1 [file DataSheet_1.pdf]

Supplemental Figure

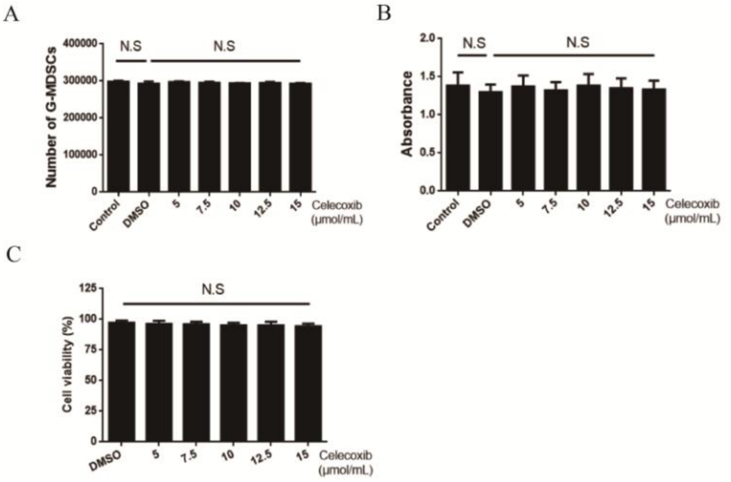

Figure S1. The effect of celecoxib on number and cell viability of G-MDSCs.

$3\times10^{\wedge5}$ G-MDSCs were inoculated into 96 well plates. Then, different concentrations of celecoxib were added into plates. After 12h, each well was added into equivalent CCK8. After 4 hours of incubation, the absorbance of each well was measured at 450nm. **(A)** The effect of celecoxib on number of G-MDSCs in each group. **(B)** The absorbance in each group. **(C)** The cell viability of G-MDSCs treated with different concentrations of celecoxib were analysed. ns: no significance.

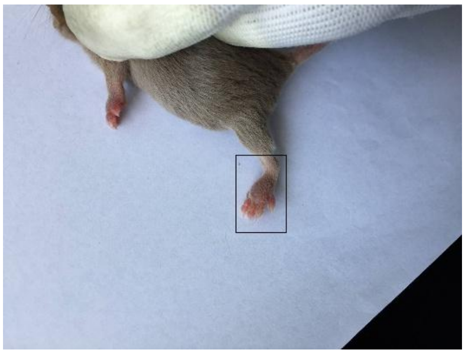

Figure S2. The original image of CIA group mice.

The hind foot of the mouse was circled in the box. After rotation, picture on the bottom left in Figure 2D could be acquired.

B10 cell

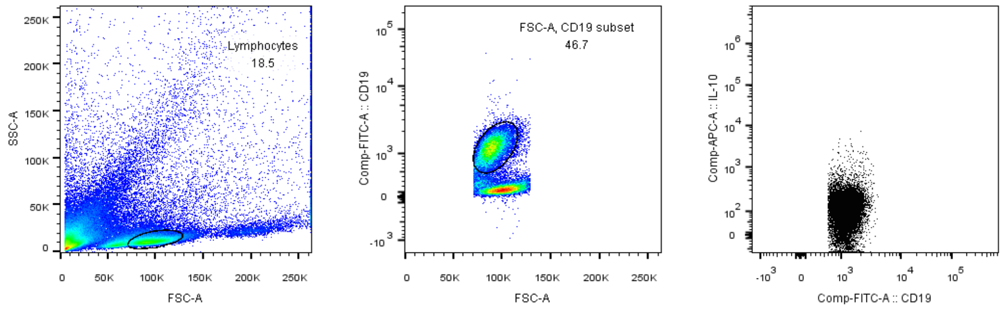

Lymphocytes were firstly gated. Then, $CD19^{+}$B cells were gated on lymphocytes. Finally, $IL\text{-}10^{+}CD19^{+}$B cells were gated on $CD19^{+}$B cells.

Plasma cell

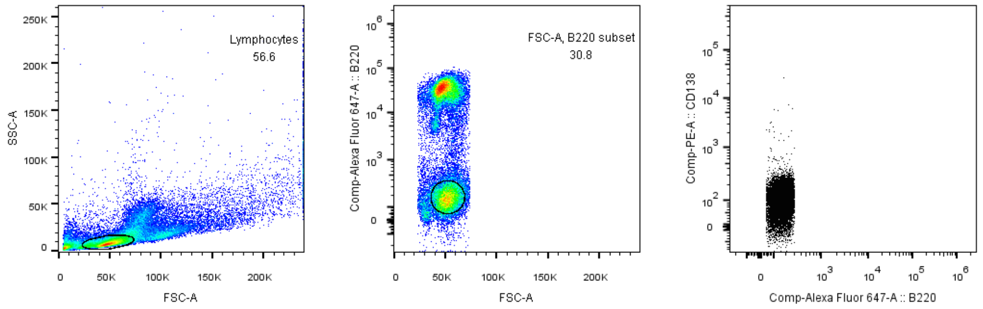

Lymphocytes were firstly gated. Then, $B220^{-}$ cells were gated on Lymphocytes. Finally, $B220^{-}CD138^{+}$ cells were gated on $B220^{-}$ cells.

Tfh cell

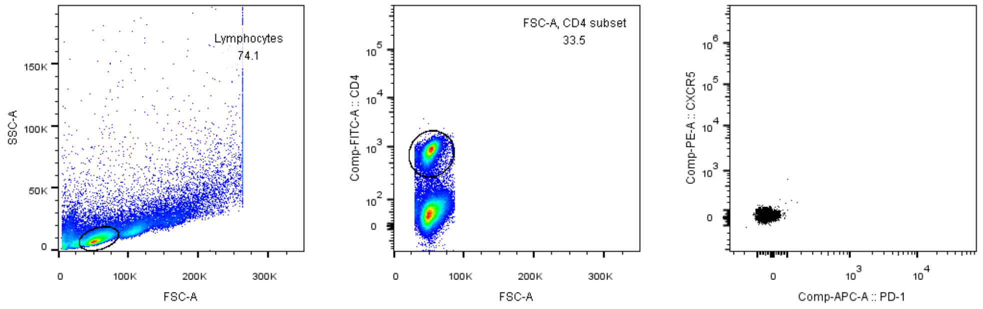

Splenocytes were stained with FITC-CD4, PE-isotype and APC-isotype.

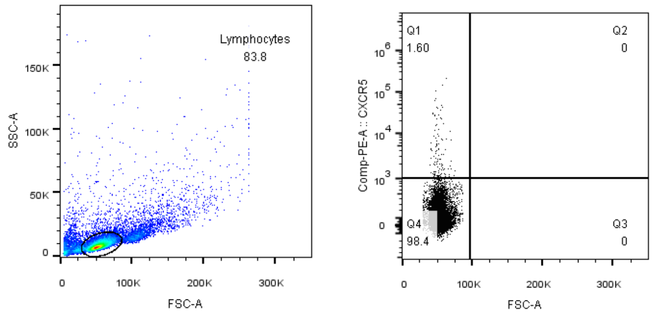

Splenocytes were stained with PE-CXCR5.

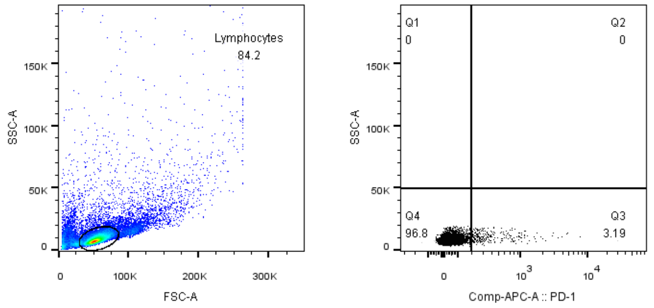

Splenocytes were stained with APC-PD-1.

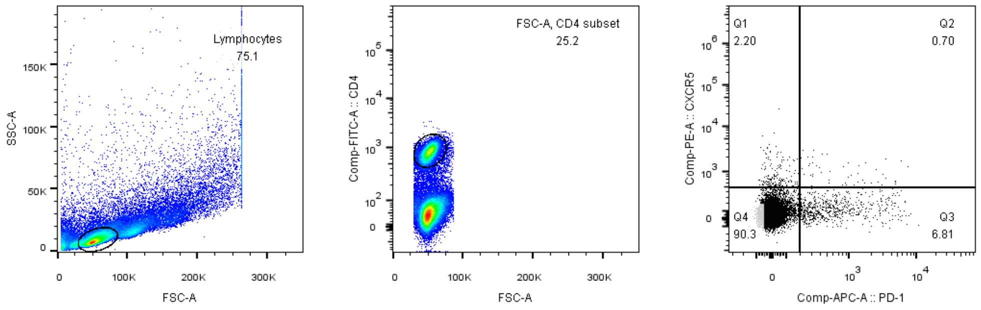

Lymphocytes were firstly gated. Then, $CD4^+$ T cells were gated on lymphocytes. Finally, $PD\text{-}1^+CXCR5^+$ cells were gated on $CD4^+$ T cells.

Figure S3. Gating on FACS plots.
